# Physiological Resilience: What Is It and How Might It Be Trained?

**DOI:** 10.1111/sms.70032

**Published:** 2025-03-02

**Authors:** Andrew M. Jones, Brett S. Kirby

**Affiliations:** ^1^ Public Health and Sport Sciences University of Exeter Medical School, Faculty of Health and Life Sciences Exeter UK; ^2^ Nike Sport Research Lab, Nike Inc Beaverton Oregon USA

**Keywords:** durability, economy, endurance, exercise, fatigue, oxygen uptake, physiology, sport

## Abstract

Physiological resilience has recently been recognized as an additional factor that influences endurance exercise performance. It has thus been incorporated into a modified, contemporary version of “the Joyner model” which acknowledges that start‐line values of V̇O_2_max, efficiency or economy, and metabolic thresholds are prone to deterioration, often with appreciable interindividual variability, during prolonged endurance exercise. The physiological underpinnings of resilience are elusive and sports physiologists are presently concerned with developing practical testing protocols which reflect an athlete's resilience characteristics. It is also important to consider why some athletes are more resilient than others and whether resilience can be enhanced—and, if so, which training programs or specific training sessions might stimulate its development. While data are scant, the available evidence suggests that training consistency and the accumulation of relatively large volumes of training over the longer‐term (i.e., several years) might promote resilience. The inclusion of regular prolonged exercise sessions within a training program, especially when these include bouts of high‐intensity exercise at race pace or above or a progressive increase in intensity in the face of developing fatigue, might also represent an effective means of enhancing resilience. Finally, resistance training, especially heavy strength and plyometric training, appears to have positive effects on resilience. Considerations of training for resilience, alongside other more established physiological determinants of performance, will likely be important in the long‐term development of successful endurance athletes.

## 1 What Is Physiological Resilience?

In recent years, the concept of physiological resilience, or durability, has become increasingly prominent in the world of endurance sport [1, 2, 3]. This concept, which echoes earlier ideas concerning “endurance capacity,” reflects an athlete's fatigue resistance during long‐duration competition. While the term “durability” was first coined by Maunder and colleagues in 2021 [3], arguably the (re‐)emergence of the concept was stimulated by a series of papers by Clark et al. [4, 5, 6] which were connected to the study of elite marathon runners as part of Nike's 2017 “Breaking 2” marathon project [2]. In the opinion of the present authors, “resilience,” defined as “the ability to resist functional decline following acute and/or chronic stressors,” provides an accurate definition of the concept.

The physiological determinants of endurance exercise performance were schematized by Joyner [7] (see also ref. [8]). The “Joyner model” has proven to be useful in understanding the interaction of the three “pillars” of aerobic exercise physiology, namely V̇O_2_max, economy, or efficiency of movement, and the sustainable fraction of the V̇O_2_max for a given race distance (which is, in turn, linked to metabolic/lactate threshold phenomena), in the determination of endurance exercise performance. This is encompassed in the following equation:

(1) $$\begin{array}{c} \text{Marathon}\;\text{speed}=\dot{V}O_{2}\max\;•\;\text{sustainable}\;\text{fraction of}\;\dot{V}O_{2}\max\;\left(\times 60\right)/\text{running}\;\text{economy} \end{array}$$

For example, in the context of elite‐level marathon running, an athlete with a V̇O_2_max of 75 mL/kg/min and a steady‐state O_2_ cost (i.e., running economy) of 200 mL/kg/km who is able to sustain 85% of V̇O_2_max for 42.2 km would be predicted to be capable of completing a marathon in 2:12:24 (h:min:s; i.e., 75 × 0.85 × 60/200 = 19.125 km/h and 42.2/19.125 = 2.2065 h). An athlete with somewhat different physiological characteristics (e.g., 80 mL/kg/min, 180 mL/kg/km and 80%) would have a different estimation for the best possible marathon time (i.e., 1:58:41). The Joyner model therefore explains how the same performance outcome can be achieved through different combinations of these physiological attributes. Note that while exercise performance is also influenced by a contribution from anaerobic energy metabolism, this contribution to energy turnover during the marathon is quantitatively and relatively small and is considered to be negligible in the model.

**FIGURE 1:**
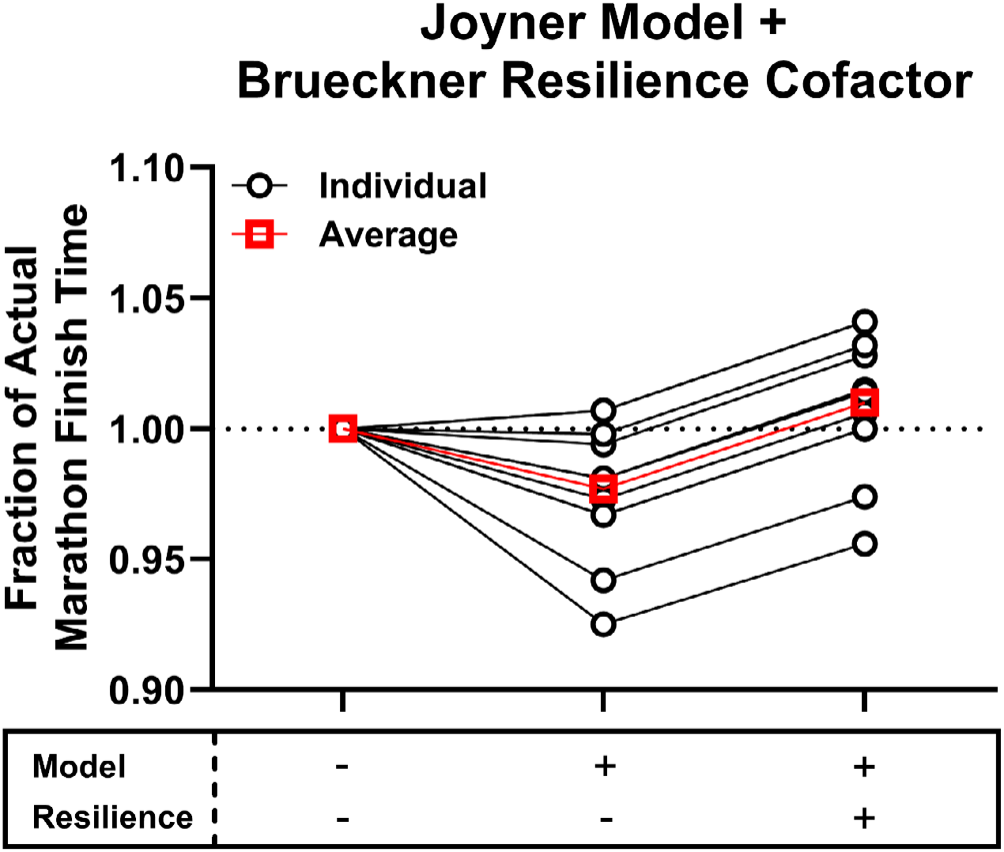
Actual marathon finish times compared against prediction estimates using the Joyner model [7] alone or the Joyner model with the incorporation of a generalized physiological resilience factor based on running economy data from Brueckner et al. [9]. Data are from 11 elite marathon runners [2] who had recent marathon performances. Model estimates of marathon finish times are normalized to the fraction of actual marathon finish times to better visualize the effect of adding a physiological resilience component to the model. Note that, on average, the Joyner model tends to overestimate best marathon performance and that adding a physiological resilience factor improves the model accuracy (with the estimate being not significantly different from actual performance). Note also, however, appreciable inter‐individual variability in the model accuracy indicating that the resilience factor should be determined on an individual basis. Individual data are presented in open circles and mean data are presented in red open squares.

Nike's Breaking 2 marathon project involved the initial physiological evaluation of 17 of the world's best distance runners [2], from whom three athletes were selected who were deemed to be potentially capable of breaking 2 h for the marathon. History shows that of the three athletes selected, only one achieved the feat, with Eliud Kipchoge first running 2:00:25 in the Breaking 2 event in Monza in 2017 and then 1:59:40 during the Ineos 159 Challenge event in 2019. When the physiological variables measured in the athletes were entered into the Joyner model, the predicted marathon time was closely correlated with the athletes' personal best marathon times. However, the Joyner model tended to slightly overestimate the athletes' best performances, with only Kipchoge proving capable of achieving the predicted best possible time (Figure 1). These considerations have led to the proposition that an additional factor, which helps to explain Kipchoge's dominance in marathon running since 2015, should be included in the Joyner model to enhance accuracy in performance prediction [1]. Indeed, it has been suggested that resilience represents the fourth physiological determinant—in addition to the three already included in the Joyner model—of endurance exercise performance [1]. Specifically, resilience is a term that accounts for the deterioration over time of the “fresh state” or “starting line” values of V̇O_2_max, economy, and metabolic/lactate thresholds during endurance competition.

**FIGURE 2:**
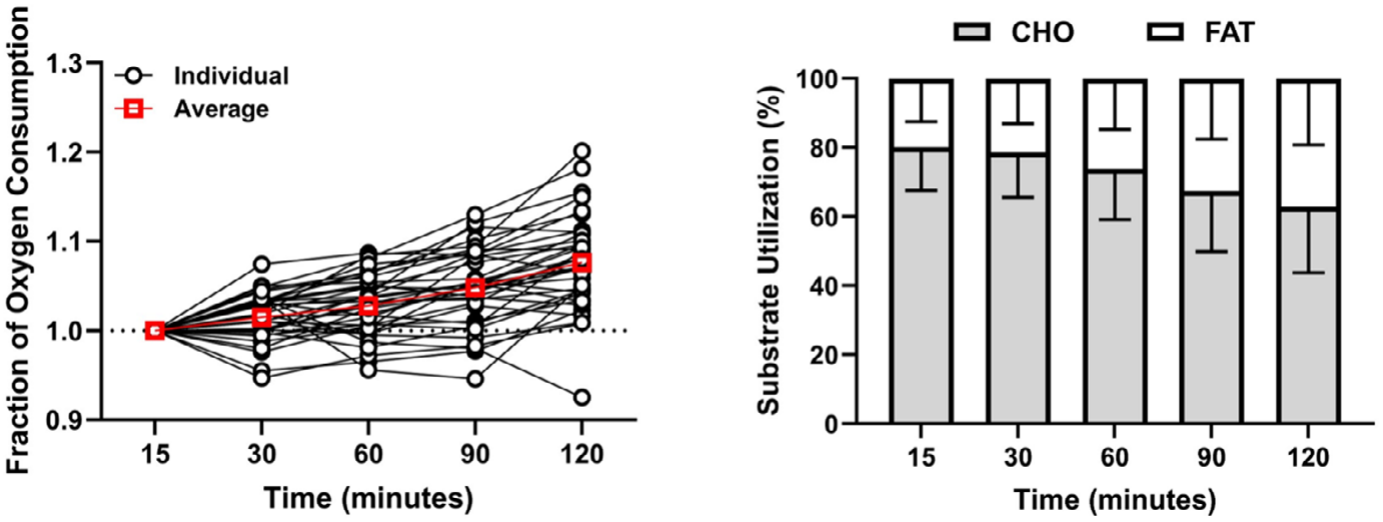
Change in oxygen consumption (V̇O_2_) over time during 2 h of heavy‐intensity cycle ergometry is shown in Panel A. Data from 36 participants from three prior studies [4, 5, 6] are shown and are normalized to the V̇O_2_ at minute 15 to depict the influence of time and individual variability on the loss of efficiency over time. Individual data are presented in open circles, and mean data are presented in red open squares. The change in proportional utilization of carbohydrate (CHO; gray color) and fat (FAT; white color) in the same participants is shown in Panel B. It can be calculated that approximately 30% of the increased V̇O_2_ during 2 h of heavy exercise can be attributed to a change in substrate utilization due to a fall in RER from approximately 0.91 to 0.84.

Some deterioration of the traditional aerobic fitness parameters during endurance competition is, of course, to be expected, and Joyner [7] mentions possible “drift” in his 1991 paper. For example, the O_2_ cost of exercising at the same speed or power is likely to increase over time [9] consequent to an increasing rate of fat compared to carbohydrate utilization [10] (Figure 2), with the former requiring a higher rate of O_2_ consumption to support a given rate of ATP turnover [11]. In addition, glycogen depletion in, or damage to, muscle fibers that are recruited early during endurance competition would lead to the activation of muscle fibers that are positioned higher in the recruitment hierarchy [12, 13] and that are likely to have a higher ATP and/or O_2_ cost of contraction [13, 14, 15]. Fatigue, per se, might also adversely impact muscle efficiency irrespective of fiber type [16]. As fatigue develops during prolonged and/or intense endurance exercise, an athlete's technique and biomechanics may also change [17], particularly in running, and this has the potential to contribute to an increased O_2_ cost of locomotion [18]. An increased O_2_ cost during prolonged exercise will therefore reflect changes in both metabolic and mechanical efficiency. Physiological variables that are sensitive to O_2_ delivery, such as V̇O_2_max and the metabolic/lactate thresholds, may also be expected to decline over time during endurance exercise as blood volume declines due to sweat loss and competition for blood flow between skeletal muscle and skin increases, in the face of elevated core temperature [19]. A simultaneous decline in V̇O_2_max and loss of efficiency during prolonged endurance exercise would conflate to result in a marked increase in the relative intensity of exercise. For example, a 7% increase in O_2_ cost (from, say, 3.00–3.21 L/min) along with a 3% reduction in V̇O_2_max (from, say, 4.0–3.88 L/min) would increase the fractional utilization of V̇O_2_max from 75% to 83%.

While such effects are indeed expected and have been reported previously, the work of Clark and colleagues [4, 5, 6] demonstrated that the extent of physiological deterioration, or fatigue resistance, during endurance exercise exhibited marked interindividual variability. Specifically, the critical power (CP), which represents the boundary separating the heavy and severe exercise intensity domains within which a metabolic steady state can, or cannot, be achieved [20], as estimated from a 3‐min all‐out cycle exercise test [21], was reduced by approximately 10% at the group mean level when measured immediately following 2 h of heavy‐intensity exercise [4, 5, 6]. However, despite the participants exercising at ostensibly the same relative intensity, the extent of the decline in CP varied between individuals, ranging from as little as 1% to as much as 33% [1]. The magnitude of the deterioration in CP was not correlated with any “baseline” physiological characteristic such as V̇O_2_max or fresh CP. These observations led to the proposition that physiological resilience represents an independent physiological determinant of endurance exercise performance—specifically, the performance outcome depends not just on the physiological characteristics of the athlete when they toe the start line at the beginning of a race [7] but also on the extent to which those characteristics decay during the event [1]. Essentially, the three aerobic pillars of the Joyner model need to be considered as dynamic, rather than static, entities [1, 2, 3].

The critical speed (CS) in running is analogous to the CP in cycling. The importance of resilience during long‐distance running can be illustrated by considering a marathon runner who sets out at a speed that is apparently sustainable for the 42.2 km distance, perhaps 92% of CS and therefore within the heavy‐intensity domain. If the CS were to decline by 10% over the course of the marathon, then the runner's speed would represent a progressively greater fraction of CS with each passing kilometer and would feel, and indeed be, less “sustainable.” At some point, it would even be possible for the CS to drop below the athlete's race speed and for the athlete to enter the severe‐intensity domain. However, in this scenario, the athlete's ability to continue would be extremely limited, and a more likely outcome is that the athlete's speed would be reduced commensurately with the fall in CS.

Clark et al. [5] reported that the fall in CP over 2 h of heavy‐intensity cycling was not linear but rather tended to accelerate markedly beyond approximately 80 min of exercise. This encroachment of the falling CS on the desired race pace is consistent with the sensation of “hitting the wall” (i.e., finding it increasingly difficult and/or being unable to maintain a previously comfortable speed) during the marathon. The fall in CP or CS during prolonged endurance exercise may be explained by increasing difficulty in matching muscle O_2_ supply to O_2_ requirement; CP and CS are sensitive to O_2_ availability [22] and a fall in V̇O_2_max consequent to cardiovascular limitation may be mirrored by a fall in CP or CS. The fall in CP or CS could also occur consequent to an increasing O_2_ cost for a given power or speed. This is because CP and CS represent the highest V̇O_2_ that can be maintained in a steady‐state, and the speed or power associated with this V̇O_2_ is derived from the V̇O_2_‐speed or V̇O_2_‐power relationship [23]. An increased V̇O_2_ for a given power or speed (i.e., loss of efficiency; Figure 2) would therefore necessarily lower the CP or CS. It is possible that, during an event such as a marathon, depending on the environmental conditions, O_2_ supply to muscle becomes increasingly limited over time and also that running economy deteriorates due to the factors mentioned earlier, with the net result being a significantly lower CS. It should be noted here that in the studies of Clark et al. [4, 5, 6], in addition to the fall in CP, the curvature constant of the power‐duration relationship, which is known as W' in cycling and D′ in running and represents a fixed amount of energy (kJ) or distance (m) available to the athlete when the CP or CS is exceeded [20], was also significantly impaired by prolonged heavy‐intensity exercise. Practically, this would impact performance by limiting the flexibility of an athlete's pace around CS, making it more difficult to initiate or respond to surges in speed for tactical reasons, to assume an effective position at feeding stations, or in any sprint finish [24]. With regard to “domain slip” during prolonged endurance exercise, it has also been shown that 2 h of exercise in the moderate‐intensity domain (i.e., below the lactate threshold) results in an approximately 10% reduction in the lactate and ventilatory thresholds which demarcate the boundary between moderate and heavy‐intensity exercise [25]. These results are consistent with Krustrup et al. [13] who showed that a protocol designed to elicit muscle glycogen depletion resulted in type II fiber recruitment and a greater O_2_ cost during moderate‐intensity cycling. Transitions between exercise intensity domains during prolonged exercise (i.e., moderate to heavy, or heavy to severe, with their attendant differences in physiological demands) have important implications for understanding the fatigue process and will also impact the accuracy of exercise or training prescription [1, 3].

The physiological determinants of resilience are, at present, uncertain but are likely to overlap with the physiological factors that are associated with endurance performance or fatigue resistance more generally. For example, a high proportion of type I muscle fibers, along with high mitochondrial volume, oxidative capacity, and capillary density, would enable a given work rate to be performed almost wholly aerobically and thus sustainably, with minimal accumulation of metabolites linked with fatigue (Pi, H+) and limited requirement to recruit less‐efficient type II muscle fibers. Fatigue during long endurance exercise is closely associated with muscle glycogen availability [26] and therefore factors related to substrate metabolism are likely also to be of importance in resilience. These may include the capacity to store a significant quantity of glycogen, the possession of a high rate of fat utilization that would spare the finite muscle and liver glycogen stores, and the ability to digest and utilize exogenously supplied carbohydrate during exercise [27]. With regard to the latter point, Clark et al. [5] showed that 60 g/h of carbohydrate ingestion during 2 h of heavy‐intensity exercise enhanced resilience, as reflected in a lesser fall in CP compared to a placebo condition. As mentioned earlier, the metabolism of free fatty acids requires more O_2_ for a given rate of ATP resynthesis compared to the metabolism of carbohydrate [11]. However, while a greater contribution of fat metabolism to energy supply during endurance exercise might marginally reduce efficiency, this may be offset by greater resilience if glycogen stores are better preserved. Further research is required to better understand these metabolic interactions in relation to different endurance exercise challenges.

In running, anthropometric factors which, in turn, influence biomechanics likely have an important bearing on resilience. Specifically, stiffer musculotendinous structures likely enhance elastic energy return during the stretch‐shortening cycle [28, 29] and may improve fatigue resistance during endurance running. East African athletes, who typically dominate long‐distance running events at the elite level, in addition to having small and light frames, tend to have relatively long shanks relative to the thigh, along with long Achilles tendons [30]. While running economy, as measured during short steady‐state running bouts, may be slightly better in African compared to Caucasian distance runners [31, 32, 33], whether the East Africans better maintain running economy during long‐distance races, and therefore have better resilience and hence performance, remains an untested hypothesis. The recent introduction of a new type of running “super shoe” which includes super‐resilient foam and a carbon plate, has coincided with numerous world records and course record performances in distance running events [34]. It is tempting to speculate that this improvement may be linked not just to improved running economy, as has been reported in short laboratory tests [35, 36, 37], but also to improved resilience (i.e., less drift in O_2_ cost during long‐duration exercise) which may be in part linked to reduced muscle damage due to improved cushioning [36, 38].

The putative mechanisms outlined above have focused on *physiologica*l resilience. The studies by Clark et al. [4, 5, 6] showed that indices of aerobic physiological function, as well as performance capacity, declined, with significant interindividual differences, following prolonged heavy‐intensity exercise. The participants in these studies were highly motivated, experienced with, and tolerant of the pain and discomfort associated with such exercise, and they completed posttrial all‐out exercise tests that satisfied the criteria for maximal effort. In this regard, resilience has a genuine physiological component. However, this is not to detract from the relevance of psychological factors in resilience, and there is likely a psycho‐physiological interaction that ultimately determines performance [39]. Lack of motivation or poor tolerance of discomfort will clearly result in poor physiological and performance outcomes [40]. It should be noted that the participants in the Clark et al. studies [4, 5, 6] were not elite athletes, and so caution is required when translating the results to more highly trained populations and inferring performance limitations.

The emergence of resilience as an independent variable that can impact endurance exercise performance creates a challenge for sports scientists because resilience is not a construct that has been measured historically, and no “off‐the‐shelf” protocols exist to enable it to be assessed in a valid and reliable way. However, it appears likely that evaluating athletes in a “fatigued” as well as in a “fresh” condition may add value to athlete diagnostics and prognostics. What is measured may depend on whether resilience is defined in performance or physiological terms and, if the latter, which variable is of principal interest. It may be the case that field tests or training data lend themselves better to the evaluation of resilience than do laboratory‐based tests, especially given the advent of increasingly sophisticated wearable devices. A concept that is related to resilience is the extent of “uncoupling” of internal and external workload during endurance exercise. Smyth et al. [41] showed that the uncoupling of heart rate from running speed during a marathon (i.e., increased heart rate for the same speed or decreased speed for the same heart rate) evidenced considerable inter‐individual variability in both magnitude and time of onset and differentiated performance in > 82,000 marathon finishers. While heart rate is sensitive to environmental influences and should not be used as a surrogate for V̇O_2_, these results do signpost possible field‐based methods that may prove useful for the evaluation and monitoring of resilience in endurance athletes. This may become increasingly important as the concept of resilience is investigated and applied in other exercise contexts such as shorter, high‐intensity endurance events and in team sports wherein superior player resilience could underpin better mental and physical performance, especially in the final quarter when the outcome of matches is often decided.

## 2 How Might Physiological Resilience Be Trained?

Given the potential importance of resilience to endurance exercise performance, athletes, coaches, and sports scientists will be interested in interventions, including training, that might enhance it. At present, however, evidence is limited concerning the training programs or specific training sessions that might stimulate physiological adaptations that improve resilience, and so it is necessary to draw inferences from the training programs of athletes that exhibit resilience and to rely to some extent on anecdote and conjecture.

The contribution of nature (genetics) vs. nurture (training) in endurance exercise physiology and performance has been debated for decades, and the extent to which resilience, per se, is innate or may be trainable is unclear. There is at least some evidence that endurance training, in general, can enhance resilience. Unhjem [42] reported that, compared to “active adults” (V̇O_2_max of approximately 50 mL/kg/min), trained runners (V̇O_2_max of approximately 64 mL/kg/min) exhibited a significantly smaller increase in O_2_ cost and a significantly smaller reduction in V̇O_2_max during a 1‐h treadmill run performed at a speed designed to require 70% V̇O_2_max. These differences in the changes in O_2_ cost and V̇O_2_max between the two groups resulted in the relative intensity of the exercise bout changing by 2.6% in the trained runners and by 8.3% in the active adults. It has also been shown that, when previously untrained people engage in a training program, physiological drifts during prolonged low‐intensity exercise are attenuated irrespective of whether the participants perform extensive low‐intensity training or much lower volume high‐intensity training [43]. When athletes of different performance calibre and with different training characteristics are compared, a similar pattern emerges. Zanini et al. [44] reported that when runners completed 90 min of treadmill running at the first lactate threshold, corresponding to approximately 80% V̇O_2_max, despite running at a higher speed and covering more distance, the higher performing group (best 10 km time of 31:20 ± 01:00, training volume of approximately 92 km/week) evidenced a smaller change in O_2_ cost of 2.4% compared to the lower performing group (best 10 km time of 41:50 ± 01:20, training volume of approximately 43 km/week) whose O_2_ cost changed by 4.5%. Although this study demonstrated clear separation between the two groups, it was not possible to differentiate the effects of aerobic fitness, performance status, or training volume on the resilience of running economy.

A further clue concerning factors that influence resilience comes from data that indicate that mature, experienced professional cyclists have greater resilience than highly talented younger riders. Specifically, Gallo et al. [45] showed that, when measured in a fresh (no prior exercise) condition, the maximum amount of work that could be done in both 5 and 20 min trials was barely different between top‐level professional cyclists and elite juniors. However, when the 5 and 20 min tests were completed following training rides that expended increasing amounts of energy (up to 50 kJ/kg), the test performance of the juniors fell away steeply whereas the performance of the professionals was almost unchanged compared to the fresh condition. In running, former world record holders Eliud Kipchoge and Paula Radcliffe displayed outstanding resilience in dominating the marathon for several years, often running negative splits in their fastest races. Both these athletes turned to the marathon following 10–15 years of training for track, cross‐country, and/or shorter road race events. Collectively, these observations suggest that consistent, uninterrupted, chronic endurance training might be a common denominator in athletes with superior resilience. While elite endurance athletes commonly practice high volumes of endurance training on a weekly basis [46], it might be suggested that it is the accrual of this volume over many years that facilitates the development of resilience. Exactly why this might be effective is less clear but it could feasibly involve long‐term muscle fiber type transformation, with the chronic low‐frequency stimulation of repeated training resulting in Type II to Type I fiber conversion [47, 48, 49] along with other adaptations to myocellular metabolic and contractile function [50].

When considering whether there might be specific types of endurance training sessions that trigger physiological adaptations which enhance resilience, scrutiny of the training performed by the world's best East African distance runners might again be informative [46, 51]. The available literature, and our personal experience, indicate that the training of these athletes has several characteristic features, each of which might contribute to the development of resilience as well as to the three pillars of aerobic physiology described in the Joyner model. One feature is the relatively high weekly training volume (approximately 160–240 km/week) which is maintained consistently for 12–16 weeks when preparing for a major competition. Training is performed twice on most days, with the first session being completed early in the morning, often in an overnight fasted state. There is some evidence that performing relatively low‐intensity training in a fasted state might enhance muscle metabolic adaptations to training and specifically the capacity for ß‐oxidation [52]. It has been reported that Kenyan runners have 20% higher 3‐hydroxyacyl‐CoA‐dehydrogenase (HAD) activity than Scandinavian runners despite similar citrate synthase activity in the m. vastus lateralis, suggesting that Kenyan runners have a high capacity for fatty acid oxidation [33] which may, in turn, contribute to superior endurance exercise performance [53]. The high training volumes practiced by endurance athletes in other sports (e.g., cycling, swimming, rowing, cross‐country skiing), as well as distance runners, likely necessitate that a substantial fraction of the total training volume is performed in a glycogen‐depleted state and it is possible that, over the longer term, this contributes to metabolic adaptations, including an increased capacity for fatty acid metabolism, that help build physiological resilience.

To our knowledge, to date, there are no longitudinal studies that have focused specifically on identifying the most effective training programs for enhancing resilience. Nevertheless, training studies that have been designed with other goals in mind, or observational studies which track changes in resilience as a consequence of routine training, might still provide some insight. For example, it has been reported that introducing thrice‐weekly high‐intensity interval training sessions to the training program of cyclists, while maintaining overall training volume, reduced physiological strain (V̇O_2_ and blood [lactate]) during, and improved time trial performance immediately following, a 2 h moderate‐intensity cycling bout [54]. Other studies have shown that power‐duration profiles can fluctuate over the course of a competitive cycling season, with the power profile measured in a fatigued state being more sensitive to training status than the power profile measured in a fresh state [55, 56]. These changes appear to be related to training intensity distribution, particularly the volume of moderate‐intensity training performed [55], and also to a shift toward a relatively greater reliance on carbohydrate oxidation in the fatigued state [56]. Collectively, these studies point to endurance training volume, training intensity distribution, and changes to muscle substrate metabolism as key variables that may influence physiological resilience.

Although elite athletes complete high volumes of training, with the majority performed at speeds below goal race pace, the training intensity distribution is typically pyramidal in nature, at least amongst distance runners [57, 58]. This speaks to the importance of specificity in training, with a fair proportion of training being done at goal race pace, and with some being done at faster than goal race pace. In the same way that having good running economy at race pace is more important for performance than having good running economy at other speeds, it might be suggested that building resilience at race pace might require training regularly at race pace. Example sessions include “threshold” and tempo runs as well as long aerobic intervals (e.g., 15 × 1000 m, as practiced regularly by Kipchoge).

Extended endurance training sessions, such as the “long run” in athletics, might also play a key role in developing resilience. In the work of Zanini et al. [59], when athletes were matched for V̇O_2_max and 10 km performance, it was evident that those that regularly practiced long runs of greater than 90 min were better able to preserve running economy compared to those that were less accustomed to long runs (3.1 vs. 6.0% increase in O_2_ cost) over a 90 min run at ~80% V̇O_2_max. Interestingly, a key session for Kipchoge and his training partners is the long run, often of 40 km, which is performed over hilly and rough terrain and at high altitude (2000–2400 m). Interestingly, this session is rarely performed at a consistent, “steady” pace, but rather starts at an easy pace as the athletes warm up and becomes progressively more intense as the session continues (e.g., starting at ~10 km/h and finishing at ~20 km/h). In this way, the athletes work “through the gears” and are running faster as they become more fatigued. However, while it can be speculated that a progressive long run such as this, or the inclusion of repeated bouts of higher‐intensity or race pace efforts within a long endurance training session [60], might train the physiological, biomechanical and psychological constituents of resilience, specific evidence to support this is lacking. It should be noted also that chronic exposure to a low barometric pressure environment, involving respiratory, cardiovascular, hematological and muscle metabolic adaptations to compensate for the greater difficulty in matching O_2_ supply to demand during exercise might also contribute to potentially greater resilience in high altitude natives. Whether resilience can be developed in sea level residents who sojourn at high altitude for training camps remains to be investigated. Similarly, it is interesting to speculate on the possibility that increasing red cell mass through regular training in a hot environment [61] might enhance resilience during exercise in a cool environment. However, acute exposure to environmental challenges, including hypoxia and high temperatures and/or humidity, might be expected to lead to poorer resilience consequent to changes in, for example, O_2_ availability, blood flow distribution and the rate of glycogen utilization [21, 62].

There is some evidence that different forms of resistance training may improve running economy in an unfatigued state [63] and similarly, there are suggestions that such training may protect running economy (or cycling efficiency) from deterioration during prolonged endurance exercise and/or improve performance in a fatigued state (i.e., improve resilience). For example, Ronnestad et al. [64] reported that heavy strength training, performed twice per week for 12 weeks by elite cyclists, improved performance in a 5 min time trial that was completed immediately following 3 h of moderate‐intensity exercise, compared to a control group that continued with only endurance training. Ofsteng et al. [65] also showed that a group of cross‐country skiers who added heavy strength training to their endurance training program (three times per week for 8 weeks) significantly improved time to exhaustion tests that were completed immediately after a prolonged training session (90 min at 65% V̇O_2_max) compared to a control group. Consistent with this, Zanini [59] found that, compared to a control group of well‐trained runners who persisted with only endurance training, heavy strength and plyometric training for 10 weeks resulted in a less pronounced increase in O_2_ cost during a 90 min run within the heavy‐intensity domain and a substantially improved time to exhaustion during a subsequent test at 95% V̇O_2_max.

Collectively, these studies indicate that resistance training, and specifically heavy strength training and perhaps plyometric training, may enhance physiological resilience and hence endurance performance. The mechanisms responsible for such an effect remain obscure but may presumably include more robust biomechanical form that is less likely to break down under fatigue, a delayed recruitment of type II motor units, and improved elastic return during the stretch‐shortening cycling in running (see earlier discussion). While the influence of concurrent endurance and strength training on neuromuscular and metabolic adaptations is controversial, the balance of evidence suggests that interference effects are rather limited [66]. It is intriguing, however, that East African distance runners typically do not perform structured resistance training. Whether they might be even more resilient if they did is a moot point, but altering an already successful overall training program would need to be carefully considered. Adding more training to any program will reduce the amount of time an athlete can spend in recovery and risks overreaching, while replacing an endurance training session with a resistance training session has the potential to be counterproductive. Therefore, the incorporation of resistance training into an overall endurance training program must be carefully balanced to minimize risk and optimize adaptation.

Technological developments in sports apparel and, in particular, innovation in footwear have significantly impacted distance running performance since the first attempt to break the 2‐h marathon barrier in 2017. It is known that the new running shoes can acutely improve running economy when measured in short running bouts [35, 36] and it is possible that the effect is even greater in a fatigued condition such as towards the end of a marathon. This enhancement of resilience, which would positively impact performance, might occur due to the high level of cushioning in the shoe reducing the extent of muscle damage [67] and therefore retarding the rate of Type II fiber recruitment and glycogen utilization. Moreover, the use of the shoe in training appears to have resulted in athletes recovering more rapidly from sessions that would ordinarily cause significant damage and/or fatigue such that training volume and/or mean training intensity have increased. The improved distance running performances in recent years may therefore be understood in terms not only of acute effects on running economy and perhaps resilience but also of athlete conditioning.

Potential sex differences in the physiological variables which underpin sports performance have been neglected in the literature [68] and it will be important to investigate any such differences in relation to resilience [69]. There is already a hint that females might be more resilient than males. Glace et al. [70] showed that 2 h of exercise at 68% V̇O_2_max differentially affected the extent of strength loss in the knee extensors between females and males, with males experiencing significantly greater fatigue. The greater neuromuscular fatigue resistance in females was associated with a better preservation of running economy during the 2‐h run. It has also been reported that, on average, males slow more than females (15.6% vs. 11.7%) in the second half compared to the first half of a marathon [71] and that the magnitude of the decoupling between heart rate and running speed is greater in males than females [41]. It is not known, however, whether these observations are linked to inherent physiological differences or to differences in pacing strategy between the sexes, with a tendency for males to overestimate their capabilities. These results suggest that biological sex is a factor worthy of further consideration when developing training strategies for improving endurance performance.

In summary, while there is a growing appreciation of the importance of resilience in endurance exercise performance and in the physiological profiling of endurance athletes, relatively little is known about the training, including specific individual sessions and the optimal volume and intensity distribution of training within an overall program, that might enhance it. We therefore encourage the design of training studies that incorporate measurements of resilience during endurance exercise, and/or of performance following a fatiguing “pre‐load,” to better tease out the training characteristics that enhance this aspect of endurance fitness.

## 3 Perspective

**FIGURE 3:**
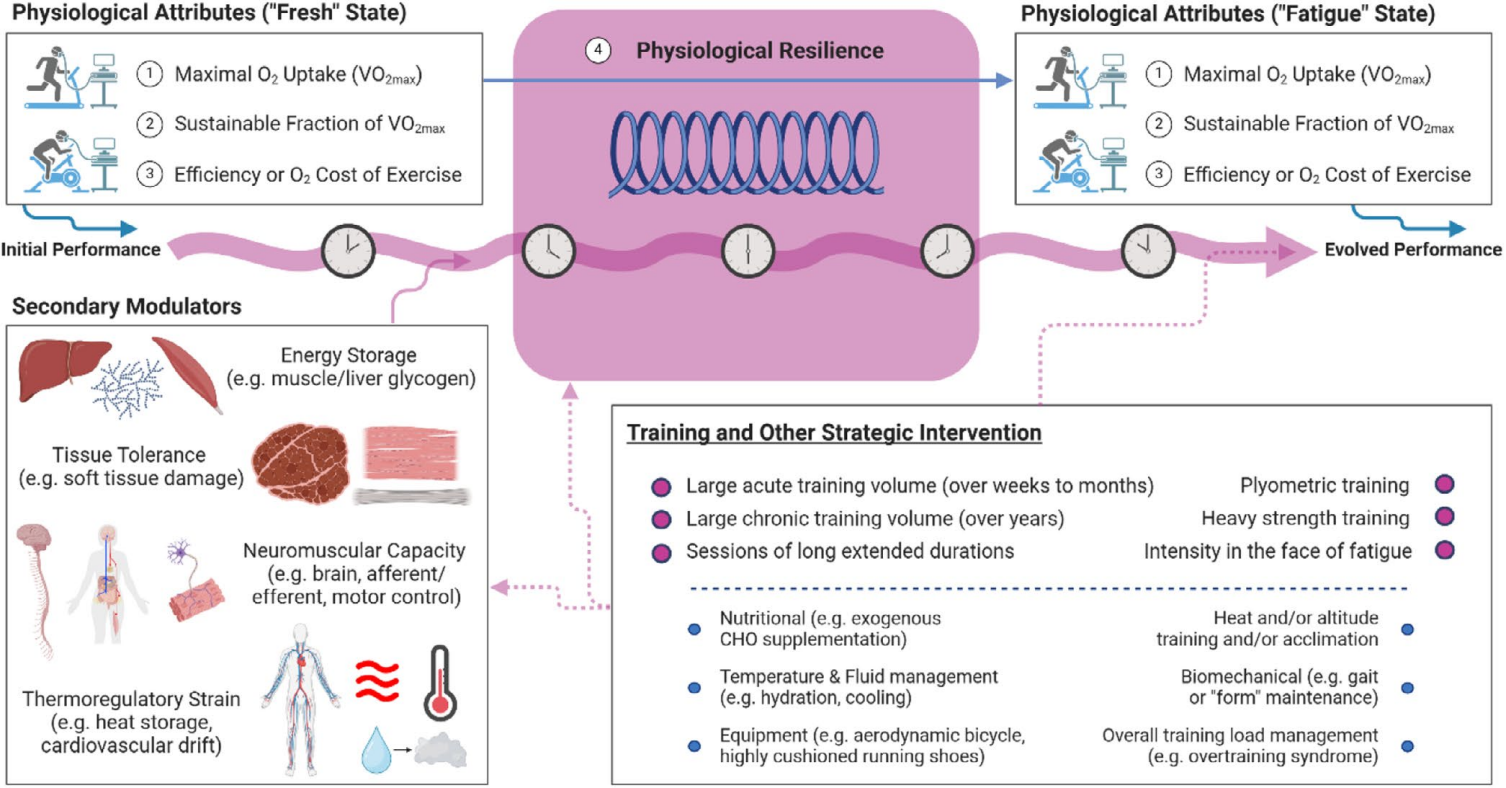
Schematic summary of the concept of physiological resilience, indicating that pre‐exercise values of the three key physiological variables integrated in the Joyner model are subject to deterioration, to a greater or lesser extent depending on individual characteristics, as endurance exercise proceeds. Some of the factors that may modulate physiological resilience, and some of the training and other interventions that may possibly enhance it, are listed. Summary graphic was created in BioRender.

Physiological resilience is an emerging concept that recognizes that the three main physiological pillars underpinning endurance exercise performance, as detailed in the so‐called Joyner model, are subject to deterioration over time as fatigue develops during prolonged exercise. The extent of this deterioration appears to be highly individual and has yet to be clearly related to any other known physiological variable, implicating resilience as an independent physiological determinant of endurance performance. The mechanisms responsible for superior resilience are unclear, practical ways to evaluate it remain to be developed, and interventions to improve it are still uncertain (Figure 3). However, with regard to training methods that might develop resilience, the available (albeit limited) evidence suggests that: any type of endurance training develops resilience compared to remaining sedentary or recreationally active; higher performing endurance athletes have greater resilience than lower performing athletes; resilience is typically better in athletes who are older or more experienced and who have a longer training history, and perhaps in those with greater training volumes and who specifically include prolonged individual training sessions within their programs. Collectively, the latter points indicate that consistent, long‐term training that allows an accumulation of high training volume might be a key stimulus for the development of physiological resilience. Specific endurance training sessions that might stimulate improvements in physiological resilience are not clearly defined, but it appears logical that specific race‐pace practice along with the inclusion of high‐intensity efforts or intensity progression within prolonged exercise bouts might be beneficial. It also appears that the inclusion of resistance training within a general endurance training program might have positive effects on resilience. While the question of what might be the “optimal” training to improve V̇O_2_max, efficiency or economy, and metabolic thresholds has stimulated sports physiologists and coaches for many decades, training for physiological resilience has emerged as a new piece of the training puzzle that should also be accounted for in the long‐term development of endurance athletes.

## Conflicts of Interest

The authors declare no conflicts of interest.

## Data Availability

Data sharing not applicable to this article as no datasets were generated or analysed during the current study.
